# A reference dataset of in-vivo human left-ventricular fiber architecture in systole and diastole

**DOI:** 10.1186/1532-429X-17-S1-Q112

**Published:** 2015-02-03

**Authors:** Constantin von Deuster, Christian T Stoeck, Martin Genet, David Atkinson, Sebastian Kozerke

**Affiliations:** 1 Division of Imaging Sciences and Biomedical Engineering, King's College London, London, UK; 2 Institute for Biomedical Engineering, University and ETH Zurich, Zurich, Switzerland; 3 Centre for Medical Imaging, University College London, London, UK

## Background

Computational cardiac modelling has been established as a valuable tool for simulating electrophysiology and electromechanics of the heart [1], with promising applications to "personalized medicine" [1]. Realistic computational models require a detailed description of left-ventricular cardiac fiber architecture. So far, non-patient-specific fiber architectures have been obtained from histology or diffusion tensor imaging (DTI) of excised post-mortem hearts. However, compared to in-vivo, ex-vivo physiological conditions including ventricular pressure and residual contractile forces deviate significantly, hence potentially impacting measured fiber metrics. The objective of this work was to obtain and make available cardiac DTI data of the in-vivo human heart with full cardiac coverage in both peak systole and mid diastole including correction for myocardial strain.

## Methods

Data from one healthy volunteer was acquired using a dual-phase dual-slice stimulated echo acquisition mode (STEAM) method [3] on a 1.5T Philips Achieva system (Philips Healthcare, Best, The Netherlands) equipped with a 5 channel cardiac receiver array. The acquisition (ECG trigger delay: 260ms & 560ms) was separated into 22 navigator-gated breath holds (ten diffusion directions, b=450s/mm^2^). The entire left ventricle was covered in both systole and diastole with a total of 12 slices with a spatial resolution of 2.2×2.2x6mm^3^.

To correct diffusion tensors for material strain, additional 3D tagging data were acquired and incorporated into diffusion tensor calculation [3]. Cardiac motion data were obtained using complementary spatial modulation of magnetization tagging (CSPAMM) [4] with spatial and temporal resolution of 3.5x7.7x7.7mm^3^ and 18ms, respectively. Diastolic and systolic diffusion tensors were mapped into a prolate spheroidal coordinate system [5] for 3D diffusion tensor field reconstruction. Data analysis was performed on the local helix elevation (helix angle α) and the deviation of the helix from circumferential structure (transverse angle β).

## Results

**Figure 1:**
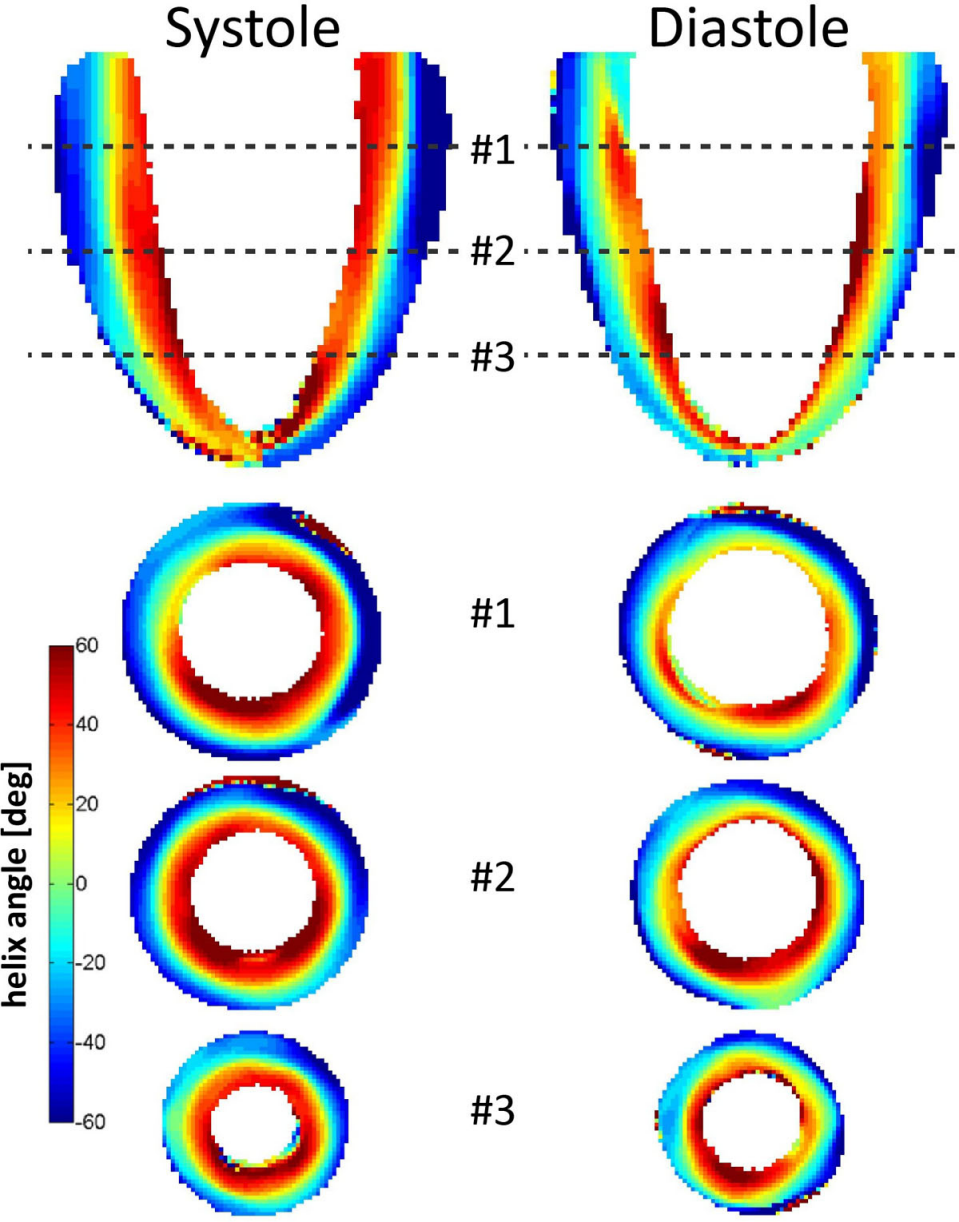
Helix angle maps for systole and diastole in long and short axis view orientation (basal, mid, apical region)

**Figure 2:**
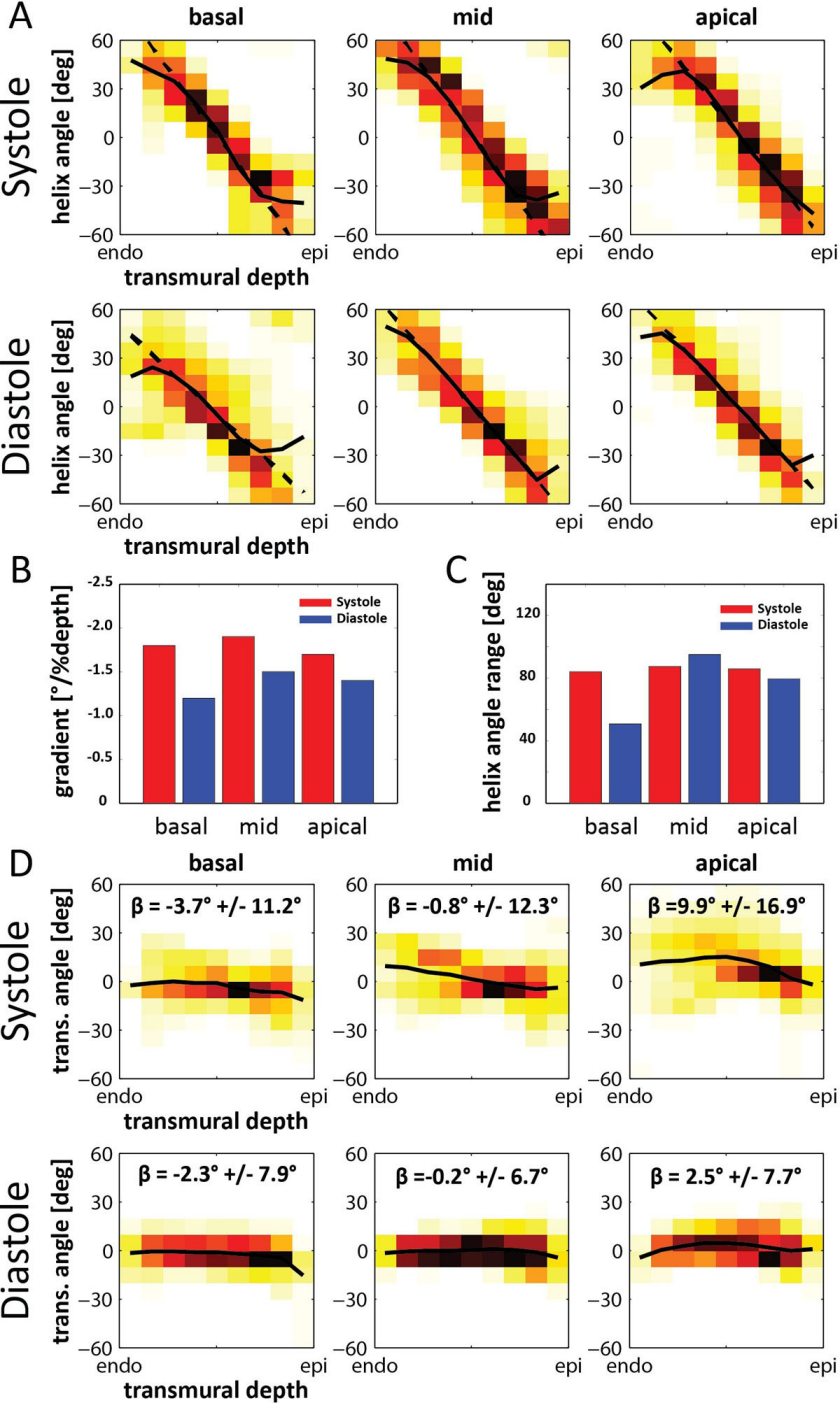
Transmural course of helix angle for basal, mid, apical region in systole and diastole (A). Slope of a linear fit model for helix angle change from endo- to epicardium (B) and corresponding helix angle range (C). Transmural course of transverse angles β with mean ± one standard deviation (D).

Data across the entire left ventricle were successfully acquired in both systole and diastole. Figure 1 shows the helix angle maps in long axis and short axis views (basal, mid, apical). The linear relation of helix angles as a function of transmural position can be seen in Figure 2A. The slope of a linear fit for all segments was found to be steeper in systole -1.8±0.1 than in diastole -1.4±0.2 with a mean helix angle range of (85.8±1.8)° in systole and (75.0±22.5)° in diastole (Figure 2B,C). Transverse angles β were closely distributed around zero degrees in both systole (1.5±14.3)° and diastole (-0.4±7.7)° (Figure 2D).

## Conclusions

The data presented here provides a comprehensive set of information on microstructure and motion and may serve as realistic input for computational modeling projects.

## Funding

This work is supported by UK EPSRC.
